# Predictive Processing in Poetic Language: Event-Related Potentials Data on Rhythmic Omissions in Metered Speech

**DOI:** 10.3389/fpsyg.2021.782765

**Published:** 2022-01-05

**Authors:** Karen Henrich, Mathias Scharinger

**Affiliations:** ^1^Department of Language and Literature, Max Planck Institute for Empirical Aesthetics, Frankfurt, Germany; ^2^Research Group Phonetics, Philipps-University of Marburg, Marburg, Germany; ^3^Center for Mind, Brain, and Behavior, Universities of Marburg and Giessen, Marburg, Germany

**Keywords:** meter, speech, prediction, trochaic preference, ERP, omission MMN

## Abstract

Predictions during language comprehension are currently discussed from many points of view. One area where predictive processing may play a particular role concerns poetic language that is regularized by meter and rhyme, thus allowing strong predictions regarding the timing and stress of individual syllables. While there is growing evidence that these prosodic regularities influence language processing, less is known about the potential influence of prosodic preferences (binary, strong-weak patterns) on neurophysiological processes. To this end, the present electroencephalogram (EEG) study examined whether the predictability of strong and weak syllables within metered speech would differ as a function of meter (trochee vs. iamb). Strong, i.e., accented positions within a foot should be more predictable than weak, i.e., unaccented positions. Our focus was on disyllabic pseudowords that solely differed between trochaic and iambic structure, with trochees providing the preferred foot in German. Methodologically, we focused on the omission Mismatch Negativity (oMMN) that is elicited when an anticipated auditory stimulus is omitted. The resulting electrophysiological brain response is particularly interesting because its elicitation does not depend on a physical stimulus. Omissions in deviant position of a passive oddball paradigm occurred at either first- or second-syllable position of the aforementioned pseudowords, resulting in a 2-by-2 design with the factors foot type and omission position. Analyses focused on the mean oMMN amplitude and latency differences across the four conditions. The result pattern was characterized by an interaction of the effects of foot type and omission position for both amplitudes and latencies. In first position, omissions resulted in larger and earlier oMMNs for trochees than for iambs. In second position, omissions resulted in larger oMMNs for iambs than for trochees, but the oMMN latency did not differ. The results suggest that omissions, particularly in initial position, are modulated by a trochaic preference in German. The preferred strong-weak pattern may have strengthened the prosodic prediction, especially for matching, trochaic stimuli, such that the violation of this prediction led to an earlier and stronger prediction error. Altogether, predictive processing seems to play a particular role in metered speech, especially if the meter is based on the preferred foot type.

## Introduction

Spoken language is based on »quasi-regular« properties, exemplified by physiological and articulatory processes such as the vibration pattern of the vocal folds or the repetitious sequence of consonants and vowels (Greenberg et al., 2003; Reetz and Jongman, 2008). It is not surprising, then, that these regularities are considered within models of speech processing that capitalize on predictions (e.g., Kutas et al., 2011; Pickering and Garrod, 2013; Schröger et al., 2015; Kuperberg and Jaeger, 2016). Predictive language processing is an umbrella term to subsume approaches that focus on context effects on all levels of the linguistic hierarchy. With the rise of frameworks related to the predictive coding theory of human brain function (Friston, 2003, 2005, 2008; Kiebel et al., 2009), these context effects were translated into prediction or expectation effects. Oftentimes, the terms prediction and expectation have been used synonymously. Here, we attempt to distinguish between the more general concept of an expectation as reflecting the anticipation of a higher-order linguistic unit, and the more concrete concept of a prediction as reflecting the temporal and content-based forecast of a specific linguistic unit. For instance, in the sentence “A salmon is a…,” the expectation is that an animate noun is following, while the specific prediction is that the word will start with the sound [f] (in “fish”).

Regularities in spoken language have a particular relation to predictions, because they allow for these predictions to be sharpened (Schröger et al., 2015; Scharinger et al., 2016). Aside from the quasi-regular properties of speech, specific forms of language use characteristically exploit these regularities. A prime candidate for such language use is poetic language, where in Western tradition, regularities hold on the level of timing (expressed in rhythm and meter) and on the level of phonological, segmental properties (expressed in assonance, consonance, alliteration, and rhyme; Jakobson, 1960; Menninghaus et al., 2017). A third level, that is also crucial for non-poetic language, concerns speech prosody, i.e., all supra-segmental properties of speech such as stress, intonation and melody. Prosodic frameworks allow to describe regular sequences of syllables on the basis of syllable weight (Nespor and Vogel, 1986; Selkirk, 1995). Here, a basic distinction has been made between the pattern of strong syllables followed by weak syllables (SW-pattern, or trochaic pattern), and the pattern of weak syllables followed by strong syllables (WS-pattern, or iambic pattern). Within the prosodic hierarchy, the combination of syllables instantiating these patterns is expressed in foot types, of which trochees and iambs are the most basic ones (Hayes, 1995).

Metrical prosodic structure in speech is of general relevance for segmentation, timing, stress, and lexical access (Jusczyk, 1999; Domahs et al., 2008, 2014; Schmidt-Kassow and Kotz, 2009; Bohn et al., 2013; Molczanow et al., 2013; Roncaglia-Denissen et al., 2013; Henrich et al., 2014; Magne et al., 2016). Violations of even subtle rhythmic preferences, as e.g., expressed by the Rhythm Rule in German, are taxing processing resources (Bohn et al., 2013; Henrich et al., 2014), while adherence to regular rhythm or meter may facilitate lexical access (Magne et al., 2007; Cason and Schön, 2012; Molczanow et al., 2013, 2019). In poetic language, regular meter and rhyme, next to further so-called »parallelistic« properties, can lead to a relative ease of processing and a simultaneous increase of aesthetic appreciation (Obermeier et al., 2013, 2015; Menninghaus et al., 2017).

Experiments investigating the neurophysiological bases of these processing consequences of regular or irregular prosody rely on event-related potentials (ERP) of the human electroencephalogram (EEG). Most of the aforementioned studies focused on a violation response that has been established in the early eighties as electrophysiological index of a semantic context effect (Kutas and Hillyard, 1980, 1984). It was then shown that semantically incongruous sentence endings elicit a distinct negative deflection in the ERP at around 400 ms after word onset. The correspondingly called N400 was initially considered to be an electrophysiological index of lexico-semantic integration, but soon received a broader interpretation in that it could also be elicited by contexts without semantic violations. In general, ease of (lexico-semantic) processing has been attributed to a decrease in N400 amplitude (Chwilla et al., 1995; Franklin et al., 2007; Lau et al., 2013).

Several studies have shown that ease of processing is not only determined by suitable semantic context but also by regular prosody (e.g., meter, see Rothermich et al., 2010; Rothermich and Kotz, 2013). A further important observation of these and similar studies is that certain prosodic patterns (such as SW vs. WS) are preferred in some, if not all languages. The SW-pattern in trochees is considered the preferred pattern or foot type in German (Wiese and Speyer, 2015). Next to preferences for a certain foot type, there also preferences as to how syllable weight determining the respective types is related to prosodic properties. Here, the so-called Iambic-Trochaic law (ITL) stipulates that rhythmic grouping strategies show a basic difference between iambs and trochees: while longer sounds or syllables tend to be assigned to group (i.e., foot) endings, louder sounds or syllables are rather assigned to group (i.e., foot) beginnings (Hay and Diehl, 2007; de la Mora et al., 2013; Crowhurst and Olivares, 2014; Crowhurst, 2020). Put differently, a typical trochee consists of a syllable with high intensity, followed by a syllable with less intensity, while a typical iamb consists of a shorter syllable followed by longer syllable. Depending on task and stimulus material, the marking of group beginnings can also be achieved by fundamental frequency (f0), or more precisely, a relative higher pitch (Crowhurst and Olivares, 2014; Crowhurst, 2020).

Electrophysiological studies focusing on the rhythmic structure of language rarely distinguish between different foot types. Of the few, Breen et al. (2019) analyzed violations of SW (trochaic) patterns as compared to WS (iambic) patterns in a reading study with EEG. Violations were realized by incongruencies between a couplet context and a target word. For trochaic violations, they found two negativities, one of which showed similarities to the N400. For iambic violations, only a positivity was elicited. This suggests that a violation of a trochaic expectancy resulted in enhanced processing effort, possibly caused by a stronger expectation in the trochaic as compared to the iambic case.

Brochard et al. (2003) were interested whether subjective accenting of identical tone sequences would yield a trochaic pattern and whether processing of stimulus changes in allegedly strong positions would differ from processing in allegedly weak positions. They employed a so-called oddball paradigm in which multiple identical tones were repeated (standards), interspersed by infrequent tones with decreased loudness in either odd- (i.e., strong) or even-numbered (i.e., weak) positions of the sequences (deviants). Oddball paradigms elicit typical ERP-responses to both deviants and standards, and an additional mismatch response to the deviant, best seen in the difference wave form between deviant ERP and standard ERP. This response is called Mismatch Negativity (MMN), typically elicited by physical stimulus changes as well as violations of higher-order regularities (Näätänen, 1995; Näätänen and Alho, 1997; Winkler, 2007). Brochard et al. (2003) demonstrated that the ERP response to deviants in odd-numbered (strong) positions (and thus, the MMN) was stronger compared to the response to deviants in even-numbered (weak) positions. Subjective accenting derived from a trochaic preference thus seems to modulate the prediction of prosodic properties (here: loudness).

The MMN has been interpreted within predictive coding frameworks, since its elicitation is thought to reflect the prediction error between the perceived stimulus and the internal model (aka the prediction), triggered by the repeating standard (Baldeweg, 2006; Winkler, 2007). As the MMN has been shown to be modulated by long-term experience with sounds in general and with speech sounds in particular (Näätänen et al., 1997; Dehaene-Lambertz et al., 2000), it is plausible to assume that prosodic preferences would similarly modulate the MMN. The study by Brochard et al. (2003) provides an important example in this respect. However, other than in the study by Brochard et al. (2003), an even more direct index of the assumed prediction error is the ERP response to a sound omission in predictive contexts. The so-called omission MMN was initially found to reflect the prediction error when a predicted tone was omitted (Tervaniemi et al., 1994; Yabe et al., 1997; Horváth et al., 2010; Salisbury, 2012), but later work showed that the omission of predicted speech sounds can also elicit the omission MMN (Bendixen et al., 2014; Scharinger et al., 2017). In the study by Bendixen et al. (2014), predictability of word-final [ks] and [ts] in the German noun “Lachs” (salmon) and “Latz” (bib) was modified by either presenting only “Lachs” or “Latz” in standard position of an oddball paradigm (predictive condition), or by randomly presenting “Lachs” and “Latz” with a 50% probability of either noun (unpredictive condition). Deviants consisted of word fragments of which the word-final consonants were omitted. The omission MMN differed between the predictive and unpredictive condition, and showed larger amplitudes in the predictive condition.

The latter study as well as previous experiments on long-term memory effects on the MMN provide the basis of our assumptions here. We hypothesize that the omission MMN between 100 and 200 post-stimulus onset (Bendixen et al., 2014; Scharinger et al., 2017) is not only modulated by segmental information, but also by prosodic information, and thus, can index violations of prosodic predictions. More concretely, on the basis of Brochard et al. (2003) we would assume that the omission of sounds in strong positions results in stronger omission responses than the omission of sounds in weak positions. We furthermore expect that the omission MMN is also sensitive to patterns of strong and weak syllables (i.e., higher-order regularities), and therefore we hypothesize that the omission of sounds in strong positions of trochaic patterns lead to the strongest omission response. Trochees can therefore instantiate the strongest metrical predictions that we intend to test by electrophysiological means, using disyllabic pseudowords with trochaic and iambic patterns and with syllable omissions occurring in either first or second position of these pseudowords. To be precise, we expect that this 2 × 2-design would show an interaction of the effects of position of omission (first syllable, second syllable) and foot type (trochee, iamb).

## Materials and Methods

### Participants

Participants were twenty native speakers of German, recruited from the participant database of the Max Planck Institute (12 females, 8 males, average age 25 ± 5 years). The sample size was based on previous studies with similar designs (Colin et al., 2009). All participants were right-handed, with scores >90% on the Edinburgh Handedness Inventory (Oldfield, 1971). None of the participants reported a history of hearing or neurological problems and participated for monetary compensation (€ 10 per hour). The study was approved by the local Ethics Committee and in accordance with the declarations of Helsinki. Prior to the experiment, participants provided written informed consent and were informed about legal aspects of the study as well as data handling policies in written and spoken form.

### Materials

**FIGURE 1:**
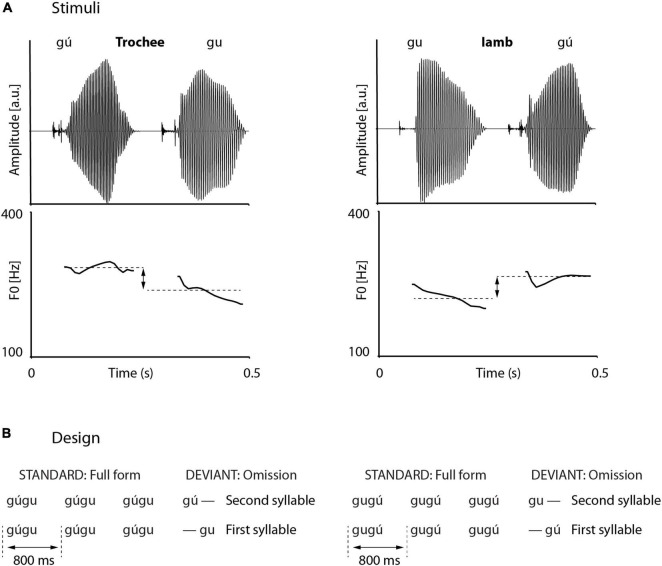
Stimulus material and design. **(A)** Pseudoword stimuli consist of cross-spliced strong (gú) and weak (gu) syllables, combined to a trochee (left) and an iamb (right). Intensities and durations are normalized, pitch tracks are similar but mean pitch height between strong and weak syllables differs by about 35 Hz. **(B)** Oddball design. Full-word deviants were interspersed by truncated deviants (omissions). Omissions could occur in word-initial or word-final syllable, and in trochees or iambs.

Trochaic and iambic stimuli were disyllabic pseudowords, starting with the voiced velar stop [g] and followed by the round, back high vowel [u], i.e., “gugu.” First, complete pseudowords were recorded in the carrier-sentence “Er soll nun gugu sagen (he shall say gugu now),” with “gugu” either pronounced with a strong initial syllable (*N* = 10) or a strong final syllable (*N* = 10). Carrier-sentences and pseudowords were spoken by a phonetically trained female speaker and recorded with 44.1 kHz temporal and 16 bit amplitude resolution in a silent recording chamber of the Max-Planck-Institute for Empirical Aesthetics in Frankfurt (Germany). From the entire set of 20 recordings, we selected those gu-syllables that had the most comparable pitch changes between strong and weak versions and showed the least difference in intensity. We decided to use stimuli that approximate typical trochaic and iambic disyllabic words without differing too much in acoustic terms, for any change of acoustic properties would modulate the omission MMN. We arrived at four syllables from one trochaic and one iambic pseudoword, of which the weak syllables had a very comparable pitch contour, differing from the strong counterparts by about 35 Hz in average pitch height. Final full-word stimuli were cross-spliced in that the original strong syllable from the selected trochaic pseudoword was combined with the weak syllable from the selected iambic pseudoword, resulting in a trochaic cross-spliced test stimulus. Vice versa, the weak syllable from the trochaic pseudoword was combined with the strong syllable of the iambic pseudoword, resulting in an iambic cross-spliced test stimulus. All syllables were trimmed to 250 ms with the phonetic software PRAAT, using the overlap-add algorithm. This was done in order to avoid MMN asymmetries that arise solely by differences in stimulus or stimulus part durations. Longer stimuli in deviant compared to standard position elicit a smaller MMN than vice versa, i.e., shorter stimuli in deviant compared to standard position (Takegata et al., 2008; Colin et al., 2009). Furthermore, all syllables were set to an internal intensity of 70 dB, corresponding to a comfortable listening level at ∼70 dB SPL when played during the experiment. Wave forms and pitch tracks of the experimental full-word stimuli are displayed in Figure 1A. Due to identical syllable durations, each disyllabic word had a duration of 500 ms.

We also analyzed the phonetic timing in the trochaic and iambic words. Due to the cross-splicing, this timing was identical across conditions. First, closure durations as measure from technical stimulus beginning until onset of the consonantal burst were 50 ms. Second, the time from the onset of the consonantal burst until the beginning of the vowels was 40 ms. In acoustic terms, this means that syllables were separated by 50 ms-pauses (corresponding to the consonantal closure durations).

Omissions were realized as syllable omissions. All omissions were created by truncating the cross-spliced pseudowords at their respective mid-points. For instance, an omission in first-syllable position of a trochee resulted in a weak syllable that originally stemmed from an iamb. In total, due to two foot types and two positions, four truncated pseudowords realized the four types of omissions.

### Design

The stimulus material was arranged in a typical oddball paradigm, where stimuli could occur in standard or deviant position, distributed over several blocks. Blocks as displayed in Figure 1B. were further split in half in order to guarantee manageable experiment times. Thus, in 2 × 4 blocks, standards consisted of disyllabic (full) pseudowords (Figure 1B) and deviants of truncated pseudowords. Truncations resulted in either first-syllable omissions or second-syllable omissions. If syllables were omitted in first position, the deviant effectively started with silence. In each block, there were 350 standards and 50 deviants (translating into 87.5% standards and 12.5% deviants). The stimulus material was pseudo-randomized, with different randomization for each participant. Constraints on randomizations were as follows: (1) minimally four consecutive standards; (2) maximally 10 consecutive standards; (3) no immediate repetition of identical standard numbers, e.g., five standards and then five standards again. The first three standards per block and standards immediately following a deviant were discarded from further analyses. The stimulus material, arranged in 2 × 4 blocks, therefore constituted a 2 × 2 design, with two levels of foot type (trochee, iamb) and two levels of omission position (first syllable, second syllable). In order to match the number of occurring strong and weak syllables, we additionally included four blocks where standards were single syllables (strong and weak syllables from trochees and iambs) and deviants were full (i.e., disyllabic) pseudowords. These four blocks were not analyzed further.

All stimuli were presented with a constant inter-stimulus interval of 300 ms. This means that inter-stimulus differences were 300 ms (measured from the end of the second syllable of one disyllabic word to the beginning of the first syllable of the next disyllabic word). This translates into a Stimulus Onset Asynchrony (SOA) of 800 ms. Note that deviants with word-initial omissions effectively resulted in an SOA of 1,050 ms measured from the beginning of the standard immediately before the deviant and the beginning of the truncated deviant syllable.

### Procedure

Stimuli were presented over open-field loudspeakers placed symmetrically 1 m in front of the participants. Participants were seated in electrically and acoustically shielded EEG-cabins. Next to the loudspeakers, a flat-screen was placed 1.2 m in front of the participants. This screen was used to display a silent movie during the passive oddball paradigm.

After EEG-setup, participants passively listened to the 12 blocks of standard-deviant trains. There was no task except the request to ignore the sounds as best as possible, while watching a silent movie (without subtitles). After each block, the experimenter allowed for a short break. In the middle of the experiment, the break was longer and the air in the EEG cabin was refreshed. Each block lasted for about 5 1/2 min; the entire experiment in the cabin about 65 min.

### Electroencephalogram Recording

Continuous EEG was recorded from 64 Ag/AgCl electrodes, arranged on a nylon cap following the extended 10–20 system (Oostenveld et al., 2011). EEG signals were amplified with a BIOSEMI ActiveTwo amplifier. Two electrodes placed left and right posterior to Cz were used as online-reference and as ground during the recording. EEG signals were recorded with a sampling rate of 500 Hz and filtered between DC and 250 Hz within the ActiveView BIOSEMI software.

### Electroencephalogram Pre-processing and Analysis

Electroencephalogram raw data were analyzed within fieldtrip (Oostenveld et al., 2011), running on Matlab (Mathworks, 2016). Electrophysiological responses were analyzed in time windows from 200 pre-stimulus onset to 800 ms post-stimulus onset. These epochs were defined on the basis of the full disyllabic words and underwent automatic artifact detection implemented within fieldtrip (Oostenveld et al., 2011). This involved detecting muscle and eye-movement (electro-oculogram) artifacts as well as epochs with amplitudes exceeding 150 μV (peak-to-peak). Automatic artifact detection led to the exclusion of individual epochs, but in no participant or condition did the exclusion rate exceed 25% of the total number of epochs (mean exclusion rate: 9.27%). Subsequently, epochs were band-pass filtered between 0.3 and 30 Hz (Hamming-window digital Butterworth filter) and re-referenced to electrodes in close proximity to the mastoids (TP9, TP10) in order to approximate a linked-mastoid reference, as is common for MMN studies (Näätänen and Alho, 1997; Schröger, 2005; Winkler, 2007). For baseline correction, the mean amplitude of the pre-stimulus window (−200 to 0 ms) was subtracted from the epoch. Responses to standards and deviants in the first-syllable and second-syllable omission conditions were then averaged separately.

### Statistical Analyses

The mismatch negativity is defined as the difference between deviant and standard responses. In order to establish electrodes and time-points at which differences between standard and deviant responses are indeed significant, we used a multi-level, non-parametric cluster statistics approach (Henry and Obleser, 2012; Strauß et al., 2014), implemented in fieldtrip (Oostenveld et al., 2011). At the first level, we calculated independent-samples *t*-tests between single-trial amplitude values for standards and single-trial amplitude values for deviants, separately for the first-syllable and the second-syllable omission conditions. We thereby obtained uncorrected by-participant *t*-values for all time points and all electrodes. These *t*-values were subsequently tested against zero using dependent-sample *t*-tests at the second, i.e., group level, of our cluster-analysis. We estimated type I-error controlled cluster significance probabilities (at *p* < 0.05) by a Monte-Carlo non-parametric permutation method with 1,000 randomizations. The resulting matrix of *t*-values (electrodes × time points) was then analyzed between 100 and 200 ms post word onset for the first-syllable omission condition, and between 350 and 450 ms post word onset for the second-syllable omission condition. These time windows represent the expected temporal location of the omission MMN, measured from stimulus onset (Bendixen et al., 2014; Scharinger et al., 2017). Within these time windows, electrodes-time point clusters were determined by neighboring electrodes and neighboring time points for which *t*-values were above the significance threshold (*p* < 0.05). In the first-syllable omission condition, this led to a cluster of 20 electrodes, showing significant standard-deviant differences between 130 and 180 ms post-stimulus onset (Figure 1). In the second-syllable omission condition, we obtained a cluster of 28 electrodes, yielding significant standard-deviant differences between 400 and 450 ms post-stimulus onset (Figure 1). Note that the latter time window corresponds to time points between 150 and 200 ms post-deviance onset. The final electrode selection for further analyses was then based on the intersection of the two electrode clusters, yielding 18 fronto-central electrodes (AF3, AF4, AF7, F1, F2, F3, F4, F5, F6, F7, FC1, FC2, FC3, FC4, FC5, FC6, FCz, and Fz).

Next, we calculated the omission MMN as difference between deviant and standard responses for the aforementioned electrodes, and in the two temporal regions as determined from the cluster statistics, separately for each participant and meter type (trochee, iamb). This resulted in mean MMN values for each participant, electrode, omission position and meter. Additionally, within the two time windows of the omission MMN, we determined the peak amplitude and the time point (latency) of this peak amplitude. Peak amplitudes were selected automatically by determining the minimum value of the deviant-standard difference in the respective time windows, and by manually inspecting the plausibility of the peaks. The automatic approach performed well, and only in three cases manual adjustment was necessary.

Cortical sources of the omission MMN were estimated using Variable Resolution Electromagnetic Tomography (VARETA; Bosch-Bayard et al., 2001; Scharinger et al., 2017). The VARETA algorithm attempts a reconstruction of cortical sources by looking for a discrete spline-interpolated solution to the EEG inverse problem. This is achieved by obtaining estimates of the spatially smoothest intracranial primary current density (PCD) distribution that is compatible with the observed scalp voltage distribution. Possible solutions are restricted to gray matter on the basis of the probabilistic brain tissue maps available from the Montreal Neurological Institute (MNI, Evans et al., 1993). First, possible sources are modeled as a pre-defined grid of voxels with 7 mm spacing. The 64 electrodes were co-registered with the average probabilistic brain atlas developed at the MNI, assuming a head radius of 85 mm. The difference ERPs of standards and deviants in the MMN time window as established by the cluster statistics were transformed into source space. Statistical parametric maps (SPMs) of the PCD estimates were then constructed based on a voxel-by-voxel Hotelling *T*^2^ test against zero (with *df* = 19).

Omission MMN mean amplitudes, peak amplitudes and latencies were then submitted to linear-effects mixed models (LMMs), calculated with the statistical software R (R Development Core Team, Vienna, Version 3.2.2). Results are reported as mixed-effects analysis of variance (ANOVAs) with *F*-values that were estimated by the lmerTest package (Kuznetsova et al., 2014), using the Satterthwaite’s method. These models used the fixed effects POSITION (omission of first syllable, omission of second syllable), FOOT TYPE (Trochee, Iamb), ELECTRODE (AF3, AF4, AF7, F1, F2, F3, F4, F5, F6, F7, FC1, FC2, FC3, FC4, FC5, FC6, FCz, and Fz) and the random effect SUBJECT in a full-factorial design (i.e., including all possible interactions).

## Results

### Amplitudes

**FIGURE 2:**
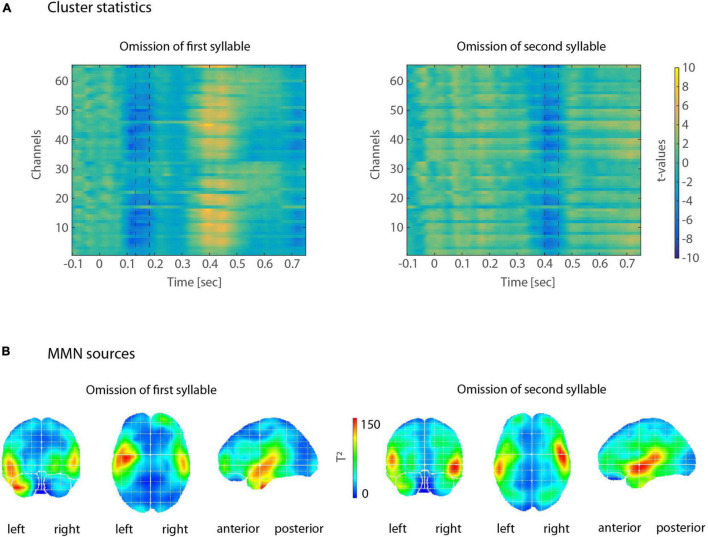
**(A)** Illustration of cluster statistics by color-coded *t*-values. Left: In the first-syllable omission condition, several electrodes showed more negative responses for deviants than for standards, between 130 and 180 ms post-stimulus onset (indicated by dashed lines). Right: In the second-syllable omission condition, more negative responses for deviants than for standards occurred between 400 and 450 ms post-stimulus onset. Color-coding of *t*-values shows warmer colors for *t*-values > 0 and cooler colors for *t*-values <0. **(B)** Results of the VARETA source reconstructions for MMNs in response to first-syllable omissions (left) and second-syllable omissions (right). Warmer colors represent higher *T*^2^-values. Sources are discernible in bilateral temporal cortices.

**FIGURE 3:**
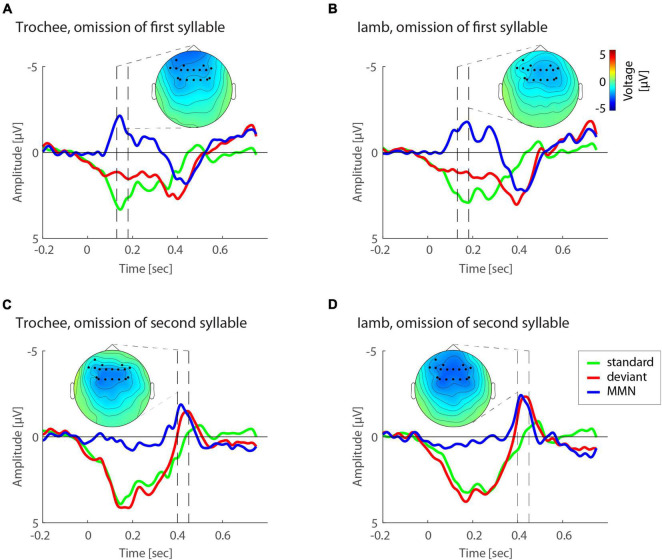
Illustration of omission MMN effects. **(A)** Event-related potentials (ERP) to standard (green) and deviant (red) responses as well as difference wave forms (blue) for trochees in the first-syllable omission condition. **(B)** ERP responses for iambs in the first-syllable omission condition. **(C)** ERP responses for trochees in the second-syllable omission condition. **(D)** ERP responses for iambs in the second-syllable omission condition. Topographies highlight the electrodes and dashed lines the temporal windows established by the cluster statistics.

Omission MMNs were reliably elicited in the typical time windows between 100 and 200 ms after deviance onset (between 100–200 ms and 350–450 ms post-stimulus onset, Figure 2). When looking at each expression of the factors POSITION (first vs. second syllable) and FOOT TYPE (trochee, iamb), topographies of omission MMNs showed typical fronto-central distributions, with sources in left and right temporal areas, including primary and secondary auditory cortex, planum temporale and parts of superior and middle temporal gyrus (Figure 3).

Statistical analyses on amplitudes are summarized in Table 1.

**TABLE 1:** Summary of mixed-effects ANOVAs on mean amplitudes and peak amplitudes.

Factor	MeanSq	NumDF	DenDF	*F*-value	*P*	Sig
**Mean amplitudes**						
POSITION	38.57	1	1,349	8.34	0.004	**
FOOT TYPE	5.48	1	1,349	1.19	0.276	n.s.
ELECTRODE	3.41	17	1,349	0.74	0.766	n.s.
POSITION × FOOT TYPE	72.16	1	1,349	15.61	0.000	***
POSITION × ELECTRODE	1.76	17	1,349	0.38	0.989	n.s.
FOOT TYPE × ELECTRODE	1.26	17	1,349	0.27	0.999	n.s.
POSITION × FOOT TYPE × ELECTRODE	1.96	17	1,349	0.42	0.981	n.s.
**First-syllable omission**						
FOOT TYPE	18.93	1	665	10.90	0.001	**
ELECTRODE	0.70	17	665	0.40	0.985	n.s.
FOOT TYPE × ELECTRODE	2.06	17	665	1.18	0.271	n.s.
**Second-syllable omission**						
FOOT TYPE	58.71	1	665	9.61	0.002	**
ELECTRODE	4.47	17	665	0.73	0.772	n.s.
FOOT TYPE × ELECTRODE	1.16	17	665	0.19	1.000	n.s.
**Peak amplitudes**						
POSITION	17.43	1	1,349	4.07	0.044	*
FOOT TYPE	5.53	1	1,349	1.29	0.256	n.s.
ELECTRODE	3.79	17	1,349	0.88	0.593	n.s.
POSITION × FOOT TYPE	43.44	1	1,349	10.14	0.001	**
POSITION × ELECTRODE	2.01	17	1,349	0.47	0.967	n.s.
FOOT TYPE × ELECTRODE	1.11	17	1,349	0.26	0.999	n.s.
POSITION × FOOT TYPE × ELECTRODE	2.32	17	1,349	0.54	0.933	n.s.
**First-syllable omission**						
FOOT TYPE	8.98	1	665	5.71	0.017	*
ELECTRODE	1.34	17	665	0.85	0.635	n.s.
FOOT TYPE × ELECTRODE	1.95	17	665	1.24	0.230	n.s.
**Second-syllable omission**						
FOOT TYPE	39.99	1	665	7.03	0.008	**
ELECTRODE	4.46	17	665	0.78	0.713	n.s.
FOOT TYPE × ELECTRODE	1.48	17	665	0.26	0.999	n.s.

*When qualified by significant interaction, first- and second-syllable omission conditions are analyzed separately.*

Significance coding: ***p < 0.001; **p < 0.01; *p < 0.05.

*n.s., not significant.*

**FIGURE 4:**
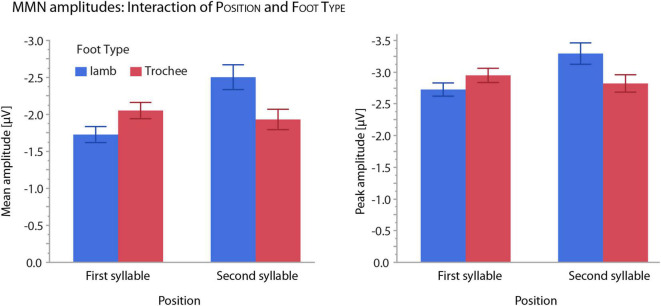
Illustration of interaction patterns between the effects of POSITION and FOOT TYPE. Left: mean amplitudes; right: peak amplitudes. Whiskers show standard errors of the mean.

For both mean and peak amplitudes, omission MMNs were larger for omissions in the second syllable (mean amplitude: −2.28 μV, peak amplitude: −3.06 μV) than in the first syllable (mean amplitude: −1.89 μV, peak amplitude: −2.84 μV). The interaction of the effects POSITION and FOOT TYPE also showed similar patterns for mean and peak amplitudes (Figure 4). Notably, in first position, trochees elicited larger MMN responses (mean amplitude: −2.05 μV, peak amplitude: −2.95 μV) than iambs (mean amplitude: −1.73 μV, peak amplitude: −2.73 μV), while in second position, iambs elicited larger MMN responses (mean amplitude: −2.50 μV, peak amplitude: −3.29 μV) than trochees (mean amplitude: −1.93 μV, peak amplitude: −2.82 μV). When the interaction was decomposed according to FOOT TYPE, Iambs [mean amplitude: *F*_(1,665)_ = 38.97, *p* < 0.001; peak amplitude: *F*_(1,665)_ = 20.57, *p* < 0.001], but not trochees (all Fs < 1, n.s.), showed higher amplitudes for omissions in second-syllable, compared to omissions in first-syllable position. All effects and interactions were independent of the electrodes.

### Latencies

Naturally, MMN latencies differed between omissions of the first and omissions of the second syllable. Overall, iambs showed longer latencies than trochees; this, however, depended on the effect of POSITION, as seen from the decomposition of the interaction of the effects of POSITION and FOOT TYPE (Table 2).

**TABLE 2:** Summary of mixed-effects ANOVAs on MMN latencies.

Factor	MeanSq	NumDF	DenDF	*F*-value	*P*	Sig
POSITION	26.21	1	1,349	129100.00	0.000	***
FOOT TYPE	0.02	1	1,349	101.44	0.000	***
ELECTRODE	0.00	17	1,349	0.63	0.874	n.s.
POSITION × FOOT TYPE	0.02	1	1,349	79.13	0.000	***
POSITION × ELECTRODE	0.00	17	1,349	1.12	0.331	n.s.
FOOT TYPE × ELECTRODE	0.00	17	1,349	0.11	1.000	n.s.
POSITION × FOOT TYPE × ELECTRODE	0.00	17	1,349	1.03	0.419	n.s.
**First-syllable omission**						
FOOT TYPE	0.04	1	665	221.02	0.000	***
ELECTRODE	0.00	17	665	1.00	0.452	n.s.
FOOT TYPE × ELECTRODE	0.00	17	665	0.53	0.939	n.s.
**Second-syllable omission**						
FOOT TYPE	0.00	1	665	0.96	0.328	n.s.
ELECTRODE	0.00	17	665	1.28	0.198	n.s.
FOOT TYPE × ELECTRODE	0.00	17	665	0.98	0.481	n.s.

*When qualified by significant interaction, first- and second-syllable omission conditions are analyzed separately.*

Significance coding: ***p < 0.001.

*n.s., not significant.*

**FIGURE 5:**
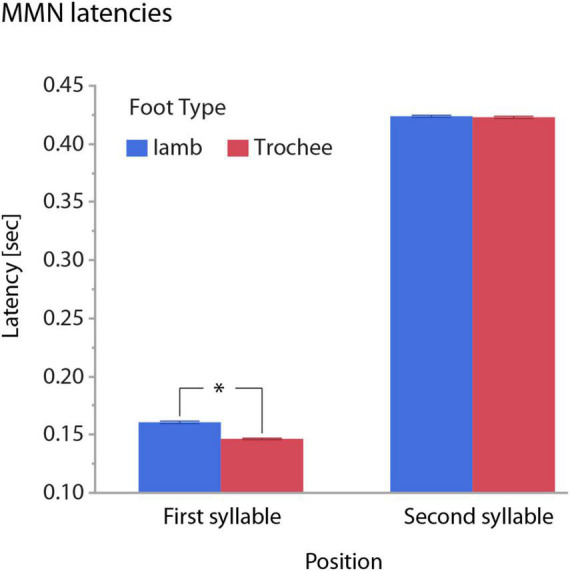
Illustration of the interaction of the effects of POSITION and FOOT TYPE on MMN latencies. Whiskers show standard errors of the mean. MMN latencies differ between trochees and iambs only in the first-syllable omission condition (significance marked by an asterisk, “*”).

The main effect of FOOT TYPE reflected on average an eight millisecond earlier omission MMN for trochees than for iambs. This effect was driven by foot type difference in the first-syllable omission condition, with significantly earlier latencies for trochees than for iambs. Here, omission MMNs occurred at 146 ms for trochees and at 161 ms for iambs. Latencies in the second-syllable omission condition did not differ between trochees and iambs (Figure 5).

## Discussion

The present study is the first omission MMN study focusing on meter perception in disyllabic speech-like structures. Its most important results on syllable omissions in regular trochaic and iambic contexts can be summarized as follows:

(a) Omissions in both first- and second-syllable position resulted in robust omission MMNs. This replicates the findings for omissions of speech sound sequences shorter than syllables (Bendixen et al., 2014; Scharinger et al., 2017) and extends the general feasibility of speech sound omissions to the level of the syllable.
(b) Omissions in second-syllable position resulted in a generally enhanced omission response compared to omissions in first-syllable position. This, however, depended on foot type and only held for iambs.
(c) Within first-syllable and second-syllable position, the main effect of FOOT TYPE indicated that first-syllable omissions resulted in larger MMNs for trochees than for iambs, and that second-syllable omissions resulted in larger MMNs for iambs than for trochees. This pattern corresponds to the weight-carrying syllable in the two foot types, with trochees consisting of a weight-carrying syllable in first position and with iambs consisting of a weight-carrying syllable in second position.
(d) The latency results suggest that an omission in first-syllable position was detected earlier for trochees than for iambs. Together with the amplitude patterns, trochees appear to imply stronger prosodic expectations, possibly caused by their preference in German (Wagner, 2012; Wiese and Speyer, 2015). The four points are further elucidated in the following sections.

### Omission Responses to Syllables

The omission MMN has been identified as prediction error response to rare omissions in tone sequences whose inter-onset intervals would not exceed a specific temporal window of integration of about 125–150 ms (Yabe et al., 1997, 1998). Subsequent work has shown that the omission response can also be elicited by speech material (Bendixen et al., 2014) and in cases where the temporal window of integration is in fact exceeded (Scharinger et al., 2017). Here, we provide evidence that omissions of syllables whose duration by far exceeded the 125–150 ms integration window can also elicit a robust omission MMN. This is the basis for our following interpretations, since we take the omission response to reflect a violation of a syllable-based prediction.

The difference between omissions in first- and second-syllable position in our experiment may—at first sight—be based on differences in temporal predictions. Second-syllable omissions are characterized by a violation of the word-internal timing. In all disyllabic pseudowords, the onset-to-onset interval of the two syllables is 250 ms. In addition to the prediction that the syllable in second position is a repeated version of the syllable in first position, there is also a strong temporal prediction that the onset of the second syllable is 250 ms after the onset of the first syllable. Temporal predictions in audition are particularly fostered by regular acoustic contexts, such as provided by oddball paradigms (Tavano et al., 2014; Auksztulewicz et al., 2018; Lumaca et al., 2019; Pinto et al., 2019). Tavano et al. (2014) and Auksztulewicz et al. (2018) explicitly refer to the need of temporal regularity for higher-order predictions, possibly supported by the brain’s dynamic sensitivity to different processing frequencies (Arnal et al., 2014), related to motor-areas (Auksztulewicz et al., 2018) or subcortical, thalamo-cerebellar circuits (Schwartze et al., 2012). In our study, omissions in first and second position may differ on the basis of temporal predictability. While second-syllable omissions may rather be sensitive to word-internal temporal regularity, first-syllable omissions should be sensitive to between-word temporal regularity. However, since word-internal as well as word-external timing is constant throughout the experiment, the interpretation of the stronger effects in second position would be that a violation of within-word temporal regularity causes a stronger prediction error than a violation of between-word temporal regularity.

### Foot Type Modulates Prosodic Predictions

A more plausible interpretation of the differences between first- and second position is based on the interaction of the effects of position and foot type. This interaction indicates that the position effect crucially depends on foot type: Only for iambs, the omission of the second syllable resulted in a larger omission MMN. That is, there is not a position effect *per se*, but rather a strong prediction of when strong syllables occur in either trochaic or iambic words. In iambic words, the strong syllable appears in second position, thus, the omission of the second syllable should result in a stronger prediction error, if the omission MMN is sensitive to prosodic properties such as syllable weight. This is supported by the results of our experiment, where indeed second-syllable omissions in iambs resulted in stronger MMNs than first-syllable position omissions. The same, complementary pattern, held for trochees: Here, omissions in first-position resulted in stronger omission MMNs than omissions in second-position. Put differently, the omission MMN is not only sensitive to syllable omissions and their temporal position, but also to the prosodic properties of these syllables, following the different foot types. This partially replicates the findings of Brochard et al. (2003) who demonstrated that the MMN depends on the subjective accenting of sound sequencing, with stronger MMNs in strong positions compared to weak positions. In our study, strong and weak positions are encoded in the acoustics of the experimental material. To this end, trochees consisted of initial syllables with a higher pitch than their final syllables, while iambs consisted of final syllables with a higher pitch than their initial syllables. In both cases, the syllables with higher pitch are likely to be interpreted as strong syllables, and the respective omission of the strong syllables resulted in a larger MMN than the omission of the corresponding weak syllables. Future studies may take this as a starting point when examining to what extent these prediction violations co-vary with higher-order, aesthetic processing. Existing studies strongly suggest an interactive effect of prosodic expectations and aesthetic appreciation (Obermeier et al., 2013, 2015; Menninghaus et al., 2015). The omission paradigm can offer a new way to quantify this correlation.

### Trochaic Preferences

Finally, when looking at the MMN latencies, our patterns of results suggest that trochees take a specific role in that the omission of their (strong) first syllable results in an earlier MMN than the omission of the (weak) first syllable of iambs. Hence, the omission of a strong syllable in first position results in a particularly salient prediction violation. Of course, it is impossible to base this effect on foot type because foot type and the position of strong syllable are confounded. To disentangle these effects, future work is necessary. However, in combination with the amplitude data, the conclusion seems warranted that trochees have a specific influence on the omission response in that this response is not only elicited at earlier latencies but also with a stronger amplitude when the strong syllable is omitted. Note that the omission of the strong syllable in iambs led to an even stronger MMN, indicating that at least the amplitude pattern does not depend on whether the syllable occurred word-initially or word-finally. Therefore, we conclude that the particular pattern elicited by trochees reflects their preferred status in German prosody (Wiese, 1996; Wagner, 2012; Wiese and Speyer, 2015). Furthermore, the latency effect in first-syllable position may also be driven by the Iambic-Trochaic Law (ITL) according to which foot beginnings are marked by higher pitch and/or higher syllable intensities, while foot endings are marked by longer syllable durations (Hay and Diehl, 2007; de la Mora et al., 2013; Crowhurst and Olivares, 2014; Crowhurst, 2020). Since we only modified pitch in our experiment, we cannot fully explore the ITL here, but suggest that earlier sensitivity to the omission of the higher-pitched syllable in trochees compared to the lower-pitched syllable in iambs is in accordance with this law. A likely articulatory explanation of this effect is that due to the respiratory cycle, word- and phrase initial syllables can be produced with higher intensities and higher pitch just because more air and more pressure is available after inhalation (see Tierney et al., 2011 for a similar explanation for song patterns in humans and non-humans).

## Conclusion

Audition benefits from local and global regularities, both temporally and phonologically (i.e., content-based). Regularities can generate strong predictions, whose violations lead to well-known electrophysiological responses. We here demonstrated the feasibility of the omission MMN to quantify foot type-based differences in prediction violations. This research can mark the starting point for further studies more concretely looking at the interplay of predictive processing and aesthetic evaluation.

## Data Availability Statement

The raw data supporting the conclusions of this article will be made available by the authors, without undue reservation.

## Ethics Statement

The studies involving human participants were reviewed and approved by the Ethics Committee of the Max-Planck-Society. The participants provided their written informed consent to participate in this study.

## Author Contributions

KH was involved in research question identification, study planning, protocol preparation, data analysis, data interpretation, and manuscript editing. MS was involved in research question identification, study planning, protocol preparation, data analysis, data interpretation, manuscript writing, editing, and reviewing. Both authors reviewed and approved the manuscript prior submission.

## Conflict of Interest

The authors declare that the research was conducted in the absence of any commercial or financial relationships that could be construed as a potential conflict of interest.

## Publisher’s Note

All claims expressed in this article are solely those of the authors and do not necessarily represent those of their affiliated organizations, or those of the publisher, the editors and the reviewers. Any product that may be evaluated in this article, or claim that may be made by its manufacturer, is not guaranteed or endorsed by the publisher.
